# UV-Induced Reduction of ACVR1C Decreases SREBP1 and ACC Expression by the Suppression of SMAD2 Phosphorylation in Normal Human Epidermal Keratinocytes

**DOI:** 10.3390/ijms22031101

**Published:** 2021-01-22

**Authors:** Yu-Dan Tian, Min Hwa Chung, Qing-Ling Quan, Dong Hun Lee, Eun Ju Kim, Jin Ho Chung

**Affiliations:** 1 Department of Biomedical Sciences, Seoul National University College of Medicine, Seoul 03080, Korea; okdanzen@snu.ac.kr; 2 Department of Dermatology, Seoul National University College of Medicine, Seoul 03080, Korea; qql0722@naver.com (Q.-L.Q.); dhlmed@hanmail.net (D.H.L.); 3 Institute of Human-Environment Interface Biology, Medical Research Center, Seoul National University, Seoul 03080, Korea; cmnmh@naver.com; 4 Department of Dermatology, Seoul National University Hospital, Seoul 03080, Korea; 5 Institute on Aging, Seoul National University, Seoul 03080, Korea

**Keywords:** activin A receptor type 1C, ultraviolet, sterol regulatory element-binding protein-1, acetyl CoA carboxylase, SMAD2 phosphorylation, epidermal lipogenesis

## Abstract

Activin A receptor type 1C (ACVR1C), a type I transforming growth factor-β (TGF-β) receptor, has been implicated in sensitive skin and psoriasis and is involved in the regulation of metabolic homeostasis as well as cell proliferation and differentiation. In this study, we identified a novel role of ACVR1C in the ultraviolet (UV)-irradiation-induced reduction of epidermal lipogenesis in human skin. UV irradiation decreased ACVR1C expression and epidermal triglyceride (TG) synthesis in human skin in vivo and in primary normal human epidermal keratinocytes (NHEK) in vitro. Lipogenic genes, including genes encoding acetyl-CoA carboxylase (ACC) and sterol regulatory element binding protein-1 (SREBP1), were significantly downregulated in UV-irradiated NHEK. ACVR1C knockdown by shRNA resulted in greater decreases in SREBP1 and ACC in response to UV irradiation. Conversely, the overexpression of ACVR1C attenuated the UV-induced decreases in SREBP1 and ACC. Further mechanistic study revealed that SMAD2 phosphorylation mediated the ACVR1C-induced lipogenic gene modulation. Taken together, a decrease in ACVR1C may cause UV-induced reductions in SREBP1 and ACC as well as epidermal TG synthesis via the suppression of SMAD2 phosphorylation. ACVR1C may be a target for preventing or treating UV-induced disruptions in lipid metabolism and associated skin disorders.

## 1. Introduction

The human epidermis is rich in lipids and is the main site of lipid synthesis in the skin, including neutral lipids, phospholipids, and sphingolipids. These lipids contribute to structural components and are involved in cell growth and differentiation and signal transduction [1]. Triglycerides (TGs) belong to the group of neutral lipids; they are a major form of energy reserve and are essential for membrane lipid synthesis [2]. The disruption of TG metabolism is related to metabolic diseases in several organs and adverse skin conditions, such as skin barrier abnormalities and skin photoaging [3,4,5,6]. TG metabolism is a dynamic process, and lipogenic and lipolytic enzymes participate in its synthesis and degradation [7]. TG synthesis is regulated by sterol regulatory element-binding proteins (SREBPs), transcription factors involved in lipid biosynthesis. SREBPs consist of three isoforms (SREBP1a, 1c, and SREBP2), and SREBP1c preferentially induces the expression of genes involved in the production of TG, including acetyl CoA carboxylase (ACC), fatty acid synthase (FAS), stearoyl-CoA desaturase (SCD), and glycerol-3-phosphate acyltransferase (GPAT) [8,9,10].

Ultraviolet (UV) radiation activates various detrimental processes, including inflammation, carcinogenesis, and premature skin aging, and contributes to skin lipid abnormalities [11,12,13]. UV irradiation also induces DNA damage, which may regulate metabolic homeostasis via transcription factors such as p53 [14]. Our previous study showed that UV decreases TG synthesis as well as the expression of related lipogenic genes in the human epidermis, suggesting that altered TG synthesis plays an important role in photoaged skin [6].

Activin A receptor type 1C (ACVR1C), also known as activin receptor-like kinase 7 (ALK7), is a type I receptor for the transforming growth factor (TGF)-β superfamily. ACVR1C is activated by various ligands, such as nodal, growth/differentiation factor 3 (GDF3), and activin B [15,16,17], phosphorylates SMAD2 and 3, and combines with SMAD4 for translocation into the nucleus, thereby regulating the transcription of target genes [18]. ACVR1C is expressed in various tissues, such as the brain, pancreas, heart, and adipose tissues, and activates downstream signaling to regulate cell proliferation, differentiation, and apoptosis [19,20,21,22]. Defects in ACVR1C are related to various diseases, including metabolic disorders, cardiac disorders, and cancers [23,24,25,26,27]. ACVR1C is expressed in human skin, and its expression is decreased in sensitive skin and psoriasis [28,29].

A previous study showed that ACVR1C is involved in lipid metabolism, which contributes to fat accumulation by suppressing lipolysis [30]. However, it is unclear whether ACVR1C affects the process of lipogenesis, particularly in the epidermis. In this study, we identified a novel role of ACVR1C in the UV-induced reduction of TG synthesis in primary normal human epidermal keratinocytes (NHEK).

## 2. Results

### 2.1. UV Irradiation Decreases the Expression of ACVR1C in the Epidermis

**Figure 1:**
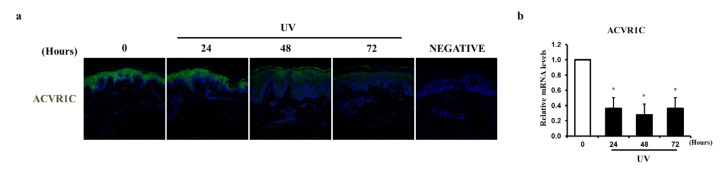
UV irradiation decreases activin A receptor type 1C (ACVR1C) expression in the human skin epidermis in vivo. Sun-protected buttock skin was irradiated with UV (two minimal erythema doses) and biopsied at the indicated times after UV irradiation. (**a**) ACVR1C protein levels were visualized by immunofluorescence staining (representative images, n = 3); (**b**) the epidermis was separated from the whole skin sample. ACVR1C mRNA levels were examined by quantitative RT-PCR (n = 4). Data are presented as means ± standard error of mean (SEM) of the expression of *ACVR1C* gene relative to 36B4. * *p* < 0.05 versus the non-irradiated control.

We previously found that the expression of ACVR1C is markedly decreased in sensitive skin triggered by various environmental factors, such as UV radiation [31]. We evaluated the effects of UV irradiation on ACVR1C expression at the protein and mRNA levels in the human epidermis. Sun-protected buttock skin was irradiated with two minimal erythema doses (MEDs) of UV and evaluated at 24, 48, and 72 h after irradiation. The epidermis was separated from the whole skin sample. ACVR1C protein expression (Figure 1a) decreased markedly after UV irradiation, as determined by immunofluorescence staining. In addition, the mRNA expression of ACVR1C (Figure 1b) was significantly reduced by UV irradiation, as analyzed by quantitative RT-PCR. These data showed that acute UV irradiation reduces the protein and mRNA levels of ACVR1C in the human epidermis in vivo.

### 2.2. UV Irradiation Decreases the Expression of ACVR1C and Lipogenic Genes in Primary NHEK

**Figure 2:**
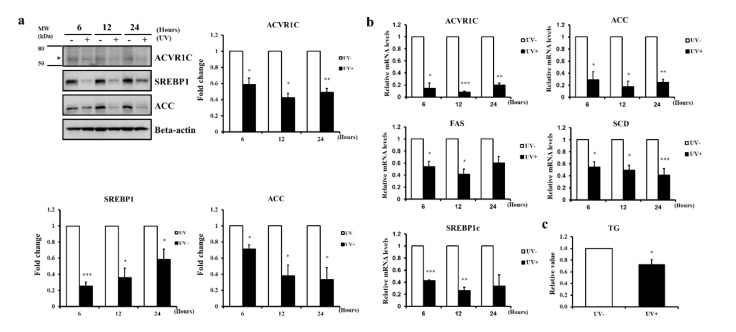
UV irradiation decreases the expression of ACVR1C as well as lipogenic genes in primary normal human epidermal keratinocytes (NHEK)**.** (**a**) Primary NHEK were irradiated with UV (75 mJ/cm^2^) and the protein levels of ACVR1C, sterol regulatory element binding protein-1 (SREBP1), and acetyl CoA carboxylase (ACC) were measured by Western blotting. β-actin was used as a loading control (n = 3). (**b**) mRNA levels of ACVR1C and lipogenic genes, such as *ACC, FAS, SCD,* and *SREBP1c*, were measured by quantitative RT-PCR after UV irradiation. Data are presented as means ± SEM of the level of each gene relative to 36B4 (n = 3); (**c**) total lipids were extracted from primary NHEK with chloroform/methanol/water (1:2:0.8, *v*/*v*/*v*). TG contents were measured by a fluorescent enzymatic method and normalized against the protein contents (n = 4). * *p* < 0.05, ** *p* < 0.01, *** *p* < 0.001 vs. the non-irradiated control.

We previously showed that UV irradiation decreases the expression of genes involved in TG synthesis in the human epidermis in vivo [6]. Consistent with these previous findings, we demonstrated that the protein and mRNA expression levels of ACVR1C and the lipogenic genes ACC and its transcriptional activator SREBP1 were significantly reduced by UV irradiation (Figure 2a,b). In addition, the mRNA expression levels of genes encoding other lipogenic enzymes regulated by SREBP1, such as fatty acid synthase (FAS) and stearoyl CoA desaturase (SCD), were significantly reduced by UV irradiation (Figure 2b). Moreover, the TG content, the final product of lipogenesis, decreased at 24 h in UV-irradiated primary NHEK (Figure 2c). These data indicated that UV irradiation reduced the levels of ACVR1C and lipogenic genes in primary NHEK, consistent with expression patterns in the epidermis in vivo.

### 2.3. Knockdown of ACVR1C Decreases the Expression of SREBP1 and ACC in Both Non-Irradiated and UV-Irradiated NHEK

**Figure 3:**
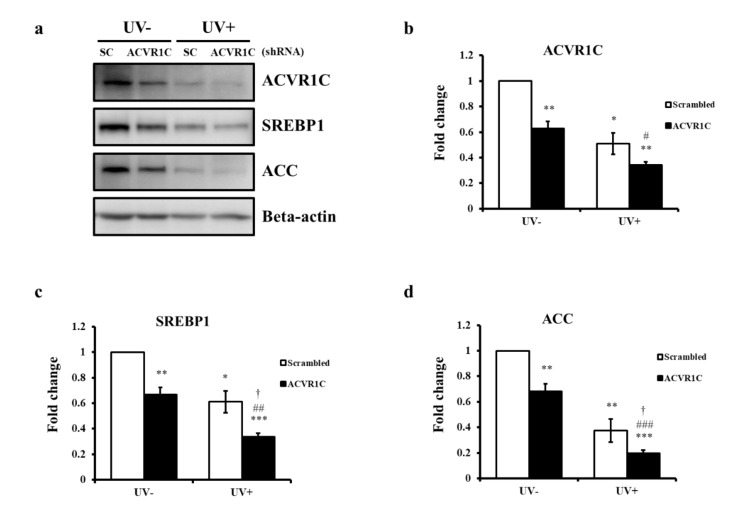
Knockdown of ACVR1C decreases the expression of SREBP1 and ACC in both non-irradiated and UV-irradiated primary NHEK. Primary NHEK were transfected with ACVR1C or scrambled shRNA and then irradiated with UV (75 mJ/cm^2^). (**a**) ACVR1C, SREBP1, and ACC protein levels were measured by Western blotting at 24 h after UV irradiation and (**b**–**d**) protein levels were quantified using ImageJ. β-actin was used as a loading control (n = 4). * *p* < 0.05, ** *p* < 0.01, *** *p* < 0.001 vs. non-irradiated scrambled shRNA; ^#^ *p* < 0.05, ^##^ *p* < 0.01, and ^###^ *p* < 0.001 vs. non-irradiated ACVR1C shRNA; ^†^ *p* < 0.05 vs. UV-irradiated scrambled shRNA.

To examine whether decreased ACVR1C is associated with reduced SREBP1 and ACC, primary NHEK cells were transfected with ACVR1C short hairpin RNA (shRNA) or a scrambled control, followed by irradiation with UV. ACVR1C protein expression levels were significantly reduced by ACVR1C shRNA in both non-irradiated and UV-irradiated cells (Figure 3a,b). The protein expression levels of SREBP1 and ACC after transfection with ACVR1C shRNA were significantly lower than those in cells treated with control shRNA in both non-irradiated and UV-irradiated cells (Figure 3a,c,d). The UV-induced decreases in SREBP1 and ACC were exacerbated in ACVR1C shRNA-treated cells (Figure 3a,b,d). These results suggested that a reduction in ACVR1C may decrease the expression of SREBP1 and ACC in both UV-irradiated and non-irradiated NHEK.

### 2.4. The Overexpression of ACVR1C Ameliorates UV-Induced Decreases in SREBP1 and ACC Protein Expression in Primary NHEK

**Figure 4:**
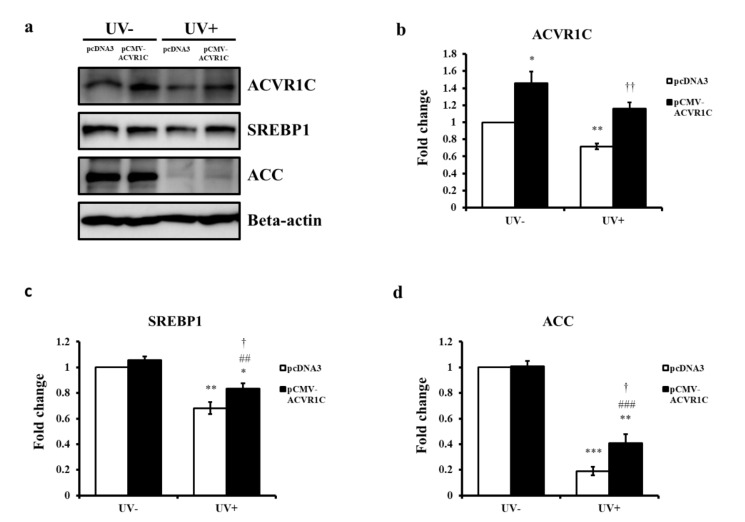
ACVR1C overexpression ameliorated UV-induced decreases in SREBP1 and ACC protein expression in primary NHEK. Primary NHEK were transfected with an expression vector containing the *ACVR1C* gene and then irradiated with UV (75 mJ/cm^2^). (**a**) ACVR1C, SREBP1, and ACC protein levels were measured by Western blotting at 24 h after UV irradiation and (**b–d**) the protein levels were quantified using ImageJ. β-actin was used as a loading control (n = 4). * *p* < 0.05, ** *p* < 0.01, and *** *p* < 0.001 vs. non-irradiated pcDNA3; ^##^ *p* < 0.01 and ^###^ *p* < 0.001 vs. non-UV-irradiated pCMV-ACVR1C; ^†^ *p* < 0.05 and ^††^ *p* < 0.01 vs. UV-irradiated pcDNA3.

To further validate the role of ACVR1C in the UV-induced reductions in SREBP1 and ACC, primary NHEK cells were transfected with an ACVR1C expression vector and then irradiated with UV. An increase in ACVR1C protein expression was confirmed at 24 h after transfection with the *ACVR1C* gene (Figure 4a,b). The protein expression levels of SREBP1 and ACC were also measured at 24 h after UV irradiation. Although the overexpression of ACVR1C did not lead to a significant change in the SREBP1 and ACC protein expression in non-irradiated cells, UV-induced reductions in SREBP1 and ACC protein expression were ameliorated in cells overexpressing ACVR1C than in control vector-transfected groups (Figure 4a,c,d). These results suggested that the upregulation of ACVR1C might prevent UV-induced reductions in SREBP1 and ACC.

### 2.5. ACVR1C Regulates the Expression of SREBP1 and ACC Protein via the Suppression of SMAD2 Phosphorylation

**Figure 5:**
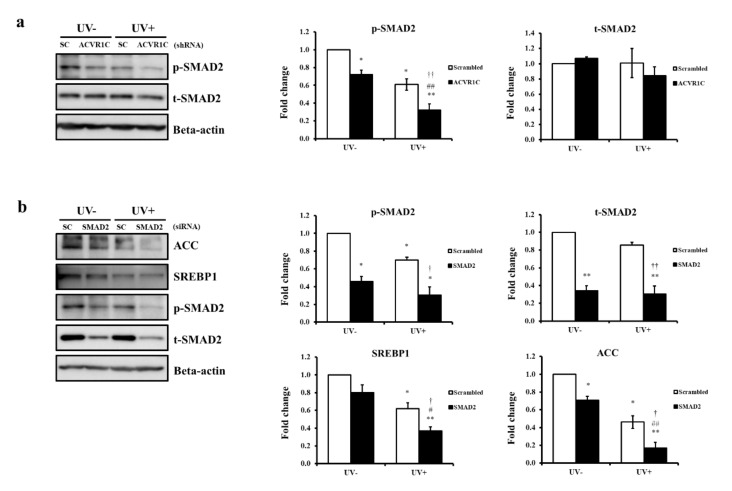
ACVR1C regulates the expression of SREBP1 and ACC protein via the suppression of the SMAD2 phosphorylation. (**a**) Primary NHEK were transfected with ACVR1C shRNA or scrambled control and then irradiated with UV (75 mJ/cm^2^). SMAD2 phosphorylation was examined at 24 h after UV irradiation (n = 3) and (**b**) primary NHEK were transfected with SMAD2 siRNA or scrambled control and then irradiated with UV (75 mJ/cm^2^). Protein levels were measured by Western blotting and quantified using ImageJ. β-actin is an equal volume of cell lysates was used as a loading control (*n* = 3). * *p* < 0.05 and ** *p* < 0.01 vs. non-irradiated scrambled siRNA; ^#^ *p* < 0.05 and ^##^ *p* < 0.01 vs. non-UV-irradiated ACVR1C shRNA or SMAD2 siRNA; ^†^ *p* < 0.05 and ^††^ *p* < 0.01 vs. UV-irradiated scrambled shRNA or scrambled siRNA.

ACVR1C controls the transcription of target genes by phosphorylating receptor-regulated SMAD2 and SMAD3 [18], and SMAD2 deficiency is related to the disruption of lipid metabolism [32]. To investigate the mechanism through which ACVR1C modulates the UV-induced reduction of SREBP1 and ACC protein expression, we examined SMAD2 phosphorylation in UV-irradiated NHEK after transfection with ACVR1C shRNA. We observed that the phosphorylation of SMAD2 decreased after UV irradiation (Figure 5). In addition, SMAD2 phosphorylation was suppressed in both non-irradiated and UV-irradiated cells by transfection with ACVR1C shRNA (Figure 5a). We also demonstrated that the levels of SREBP1 and ACC decreased in both non-irradiated and UV-irradiated groups after transfection with SMAD2 siRNA (Figure 5b). Taken together, the UV-induced reduction of ACVR1C might regulate the expression of SREBP1 and ACC protein by suppressing SMAD2 phosphorylation.

## 3. Discussion

Epidermal lipid metabolism plays an essential role in various skin functions. UV irradiation impairs epidermal lipid homeostasis. We previously demonstrated that the TG content and expression of lipogenic genes, including SREBPs, are decreased in acute UV-irradiated and photo-aged human skin [6]. In the present study, we discovered that ACVR1C, a type I TGF-β receptor, acts as a modulator of the negative effects of UV on TG synthesis. Yogosawa et al. reported that an ACVR1C deficiency upregulates lipolysis and reduces the total TG content in mature adipocytes, suggesting that ACVR1C regulates fat accumulation via the lipolysis pathway [30,33]. However, little is known about the regulatory effects of ACVR1C on TGs synthesis, another important pathway determining TG levels in the skin. In this study, we showed that UV irradiation reduces the expression of ACVR1C in the human skin and identified a novel role of ACVR1C in the UV-induced reduction of TG synthesis via the downregulation of genes involved in lipogenesis, such as SREBP1 and ACC, in primary NHEK. SREBP1c, a central regulator of TG synthesis, directly regulates ACC, which is the first enzyme in the control of TG synthesis [9].

The knockdown of ACVR1C decreased SREBP1 and ACC expression as well as the phosphorylation of the downstream mediator SMAD2. Furthermore, the knockdown of SMAD2 could downregulate SREBP1 and ACC. These data agree with previous results that showed that a SMAD2 deficiency decreases TG levels and reduces the expression of lipogenesis-associated genes, including SREBP1c [32]. UV interferes with SMAD signaling transduction in human skin, and SMAD2 phosphorylation levels are decreased in photoaged skin [34]. Our data suggested that the UV-induced reduction of ACVR1C triggers the downregulation of TG synthesis by the suppression of SMAD2 phosphorylation. Interestingly, we found that the overexpression of ACVR1C significantly restored the UV-induced reductions in SREBP1 and ACC levels only when these levels were low upon UV irradiation. These findings suggested that the upregulation of ACVR1C may regulate TG metabolism more efficiently when the epidermal lipid status is compromised by external stimuli, such as UV exposure. Andersson et al. showed that ACVR1C knockout mice exhibit reduced fat accumulation only when fed with a high-fat diet but not when fed regular chow, implying that ACVR1C signaling is relevant in stress conditions, such as under excess nutrients [35]. Additional studies are warranted to elucidate the effects of known or potential ligands of ACVR1C, such as nodal, on UV-induced changes in epidermal lipid metabolism.

Collectively, our results demonstrated that UV-induced reductions in ACVR1C cause a decrease in the expression of SREBP1 and ACC protein involved in TG synthesis via the suppression of SMAD2 phosphorylation. These findings suggest that ACVR1C could be a new target for attenuating the disruption of skin lipid metabolism by UV irradiation.

## 4. Materials and Methods

### 4.1. Human Skin Samples and UV Irradiation

Healthy human volunteers (mean age, 42.8 years; range, 40–44 years) provided sun-protected and UV-irradiated skin samples. An F75/85W/UV21 fluorescent lamp (emission range 285–350 nm, peak at 310–315 nm) was used to irradiate sun-protected buttock skin. UV-C (<290 nm) was filtered. In this study, UV irradiation included both UV-A and UV-B light. The minimal erythema dose (MED) was determined 24 h after UV irradiation. Sun-protected buttock skin was irradiated with UV (two minimal erythema doses), and non-irradiated and UV-irradiated samples were obtained by punch biopsy. The epidermis was separated from whole skin specimens following incubation at 55 °C in phosphate-buffered saline (PBS) for 2 min. This study was approved by the Institutional Review Board at Seoul National University Hospital (IRB No. 1610-097-801; 2016.11.24.), and all subjects provided written informed consent. The study was conducted according to the Principles of the Declaration of Helsinki.

### 4.2. Cell Culture and UV Irradiation

Primary NHEK were isolated from the foreskin. Cells were cultured in EpiLife™ medium containing human keratinocyte growth supplement (Thermo Fisher Scientific, Inc., Waltham, MA, USA) in a 37 °C humidified, 5% CO_2_ incubator. Before UV irradiation, cells were washed with PBS, irradiated with UV (75 mJ/cm^2^) in PBS, maintained in EpiLife™ medium, and then harvested for analysis. The UV source from the Philips TL 20W/12RS fluorescent sun lamps had an emission wavelength between 275 and 380 nm (peak, 310–315 nm). UV-C (<290 nm) was filtered. The UV irradiation intensity was measured using a UV meter (Model 585100; Waldmann Co., Villingen-Schwenningen, Germany).

### 4.3. Plasmid Transfection

A pLKO.1 transfer vector containing short hairpin RNA (shRNA) targeting sequences (#TRCN0000196633) was obtained from Sigma (St. Louis, MO, USA). Lentiviral particles were produced as described previously [36]. Scrambled shRNAs were used as controls. Primary NHEK were infected with 1 mL of medium containing lentiviral particles with 8 µg/mL polybrene (Santa Cruz Biotechnology, Dallas, CA, USA). The medium was replaced with growth medium after infection. At cells reached confluence, they were treated with UV (75 mJ/cm^2^) and harvested at 24 h. For the overexpression of ACVR1C, the plasmid expressing human ACVR1C was obtained from Sino Biological Inc. (Beijing, China). Primary NHEK were transfected with the ACVR1C human expression vector using Lipofectamine^®^ 3000 Reagent (Invitrogen, Carlsbad, CA, USA) according to the manufacturer’s instructions. Cells were treated with UV (75 mJ/cm^2^) after reaching confluence, and then harvested at 24 h.

### 4.4. RNA Interference

For the knockdown of SMAD2, cells were transiently transfected with SMAD2 siRNA or negative control siRNA (Bioneer, Daejeon, Korea) using Lipofectamine^®^ 2000 Reagent (Invitrogen, Carlsbad, CA, USA). Cells were treated with UV (75 mJ/cm^2^) after reaching confluence, and then harvested at 24 h.

### 4.5. Western Blot Analysis and Immunofluorescence Staining

Primary NHEK were homogenized, and proteins were extracted using radioimmunoprecipitation assay (RIPA) buffer (Merck Millipore, Billerica, MA, USA) containing a protease inhibitor mixture (Roche Applied Science, Penzberg, Germany) and a phosphatase inhibitor mixture (Sigma-Aldrich, St. Louis, MO, USA). Equal amounts of protein lysates were subjected to SDS-polyacrylamide gel electrophoresis (SDS-PAGE) and then blotted onto polyvinylidene difluoride (PVDF) membranes. After blocking in 5% non-fat milk diluted with Tris-buffered saline containing 0.1% Tween 20 (TBST), the membrane was incubated overnight at 4 °C with primary antibodies against ACVR1C (Atlas Antibodies, Stockholm, Sweden), SREBP1 (Santa Cruz Biotechnology, Dallas, TX, USA), ACC (Cell Signaling, Danvers, MA, USA), p-SMAD2, and t-SMAD2 (Cell Signaling, Danvers, MA, USA), and β-actin (Thermo Fisher Scientific, Inc., Waltham, MA, USA). The membranes were then washed and incubated with horseradish peroxidase-conjugated secondary antibody for 1 h at room temperature. Bands were detected using an enhanced chemiluminescence detection system (Biomax Co. Ltd., Seoul, Korea). Band density was quantified using ImageJ (NIH, Bethesda, MD, USA). Skin specimen sections (4 mm) were stained with a primary ACVR1C (MyBioSource, Inc., San Diego, CA, USA) rabbit polyclonal antibody in a humidified chamber at 4 °C for 18 h. After washing in PBS, the sections were incubated with a secondary Alexa 488-conjugated goat anti-rabbit IgG (Invitrogen, Carlsbad, CA, USA) antibody for 1 h at room temperature. Nuclei were counterstained with DAPI.

### 4.6. Real-Time and Semi-Quantitative PCR (RT-PCR)

Total RNA was prepared from the separated epidermis or NHEK using RNAiso Plus (Takara Bio Inc., Shiga, Japan), and 1 µg of total RNA was converted to complementary DNA using the First Strand cDNA Synthesis Kit (MBI Fermentas, Vilnius, Lithuania) according to the manufacturer’s instructions. To quantitatively estimate the mRNA expression level of each gene, PCR was performed on a 7500 Real-time PCR System (Applied Biosystems, Foster City, CA, USA) using SYBR Premix Ex Taq (Takara Bio Inc., Shiga, Japan) according to the manufacturer’s instructions. The sequences of primers were as follows: ACVR1C (forward, 5′- AGT CGG AGG AAT TGT TGA GGA -3′; reverse, 5′- CTC GGA GTG CTT CAC AAC TTT -3′), SREBP1c (forward, 5′- GCC ATG GAT TGC ACT TT -3′; reverse, 5′- CAA GAG AGG AGC TCA ATG -3′), ACC (forward, 5′- CCA CTT GGC TGA GCG ATT -3′; reverse, 5′- CCA GGT CCT CCA GCA GAA -3′), FAS (forward, 5′- CCG AGG AAC TCC CCT CAT -3′; reverse, 5′- GCC AGC GTC TTC CAC ACT -3′), SCD (forward, 5′- TGG AGC CAC CGC TCT TAC -3′; reverse, 5′- GCC ACG TCG GGA ATT ATG -3′), 36B4 (forward, 5′- TCG ACA ATG GCA GCA TCT AC -3′; reverse, 5′- TGA TGC AAC AGT TGG GTA GC -3′) [37].

### 4.7. Triglyceride Contents

The lipids were extracted from NHEK with chloroform/methanol/water (1:2:0.8, *v*/*v*/*v*), and triglyceride contents were determined by a fluorescent enzymatic method (Asan Pharmaceutical Co. Ltd., Seoul, Korea) and normalized to the protein content by the Bradford method (Bio-Rad, Hercules, CA, USA).

### 4.8. Statistical Analysis

Data are presented as means ± SEM. Significance was analyzed using the paired *t*-test or Student’s *t*-test. Differences were considered significant when *p* < 0.05.

## Data Availability

The data that support the findings of this study are available from the corresponding author upon reasonable request.

## Abbreviations

ACVR1C	Activin A receptor type 1C
ACC	acetyl CoA carboxylase
MED	Minimal erythema dose
NHEK	Normal human epidermal keratinocytes
shRNA	Short hairpin RNA
siRNA	Small interfering RNA
SREBP1	Sterol regulatory element-binding protein1
TG	Triglycerides
UV	Ultraviolet
